# The Promoter of the pri-miR-375 Gene Directs Expression Selectively to the Endocrine Pancreas

**DOI:** 10.1371/journal.pone.0005033

**Published:** 2009-04-03

**Authors:** Tali Avnit-Sagi, Lia Kantorovich, Sharon Kredo-Russo, Eran Hornstein, Michael D. Walker

**Affiliations:** 1 Department of Biological Chemistry, Weizmann Institute of Science, Rehovot, Israel; 2 Department of Molecular Genetics, Weizmann Institute of Science, Rehovot, Israel; University of Bremen, Germany

## Abstract

microRNAs (miRNAs) are known to play an essential role in controlling a broad range of biological processes including animal development. Accordingly, many miRNAs are expressed preferentially in one or a small number of cell types. Yet the mechanisms responsible for this selectivity are not well understood. The aim of this study was to elucidate the molecular basis of cell-specific expression of the pri-miR-375 gene, which is selectively expressed in pancreatic islets, and has been implicated both in the development of islets, and the function of mature pancreatic beta cells. An evolutionarily conserved 768 bp region of DNA upstream of the pri-miR-375 gene was linked to GFP and luciferase reporter genes, and expression monitored in transgenic mice and transfected cultured cells. Deletion and targeted mutagenesis analysis was used to evaluate the functional significance of sequence blocks within the upstream fragment. 5′-RACE analysis was used for mapping the pri-miR-375 gene transcription start site. The conserved 768 bp region was able to direct preferential expression of a GFP reporter gene to pancreatic islets in transgenic mice. Deletion analysis using a luciferase reporter gene in transfected cultured cell lines confirmed the cell specificity of the putative promoter region, and identified several key cis-elements essential for optimal activity, including E-boxes and a TATA sequence. Consistent with this, 5′-RACE analysis identified a transcription start site within this DNA region, 24 bp downstream of the TATA sequence. These studies define the promoter of the pri-miR-375 gene, and show that islet-specific expression of the pri-miR-375 gene is controlled at the transcriptional level. Detailed analysis of the transcriptional mechanisms controlling expression of miRNA genes will be essential to permit a comprehensive understanding of the complex role of miRNAs such as miR-375 in developmental processes.

## Introduction

microRNAs (miRNAs) are a class of small non-coding RNAs that regulate gene expression through post-transcriptional mechanisms [1]. Accumulating evidence indicates that miRNAs play a central role in controlling a broad range of biological activities including embryonic development, cell proliferation, metabolic homeostasis and apoptosis [2]. Mammalian genomes contain over 400 miRNA genes [3]. A significant percentage of these are nested within introns or exons of protein coding genes: in these cases, expression of the corresponding miRNAs appears to be under the transcriptional control of the host gene [4]. On the other hand, many miRNA genes are located in intergenic regions, and constitute autonomous expression units that are transcribed by RNA polymerase II [5] into capped and polyadenylated precursors (pri-miRNAs). Sequential processing by the nuclear RNase Drosha and the cytoplasmic RNase Dicer generates the ∼22 nt mature miRNA. Upon incorporation to the RISC complex, miRNAs can inhibit expression of genes by promoting degradation of mRNA, or inhibition of translation [6].

Detailed analyses of expression patterns of miRNAs has demonstrated that a significant number of miRNAs are expressed in highly selective spatial and temporal patterns [7], [8]. The molecular basis for this selectivity is not well understood, in large part because relatively little is known about the structure and function of miRNA gene promoters. As a result, it has been difficult to discriminate between transcriptional [9] and post-transcriptional regulation [10]. miR-375 is selectively expressed in pancreatic islets [8], [11]. It appears to play an important role in mature islet cell function, in part by inhibiting expression of myotrophin, a protein implicated in exocytosis [11], [12]. A role for miR-375 has also been demonstrated in pancreatic islet development, through use of morpholino oligonucleotides to reduce expression of miR-375 in developing zebrafish embryos [13]. These results are consistent with recent experiments in mice demonstrating that global inhibition of microRNA biogenesis by deletion of Dicer in embryonic pancreas leads to defects in pancreatic islet development [14].

The mechanisms controlling selective expression of protein coding genes of the endocrine pancreas have been extensively studied [15], [16]. It is well established that regulation is exerted primarily at the transcriptional level through multiple cis-elements located in the promoter regions: these activities typically involve lineage-restricted transcription factors such as Pdx-1, NeuroD1 (BETA2) and MafA [17]–[19] which interact in synergistic fashion to generate specificity [20]. The aim of the current study was to determine the molecular basis for the selective expression of the pri-miR-375 gene in pancreatic islets. Through generation of reporter plasmids, we were able to functionally characterize the pri-miR-375 promoter. The promoter shows selective activity in islets of transgenic mice and in transfected beta cells, demonstrating that regulation of cell-specific expression is mediated at the transcriptional level.

## Materials and Methods

### Ethics statement

All animal work was conducted according to relevant national and international guidelines. The experiments were approved and overseen by the Institutional Animal Care and Use Committee (IACUC) of the Weizmann Institute of Science.

### Bioinformatics

Sequence conservation was examined using the UCSC genome browser (http://genome.ucsc.edu/). Sequence alignment of genomic DNA sequences was performed using NCBI-BLAST (http://www.ncbi.nlm.nih.gov/BLAST) and software from GCG. Identification of transcription factor binding sites was performed using Genomatix suite programs MatInspector (http://www.genomatix.de) and TESS (http://www.cbil.upenn.edu/cgi-bin/tess).

### Plasmid constructions

Plasmids for promoter activity measurements were constructed using pGL3-basic vector (Promega) or the TK-luc vector [21]. The region upstream to the miR-375 gene (putative miR-375 promoter region) was generated by PCR using primers 5′ - GAAGATCTTGAGGTACATCGCAGAGGCCAG - 3′ (top) and 5′ - CATGCCATGGGGGCCGGAGCGGAAGACCC - 3′ (bottom) with template of genomic DNA. The PCR fragment was sub-cloned into pGEMTeasy vector (Promega), and ligated to pGL3-basic (SmaI site), creating construct 375a, or to TK-luc. Constructs 375d and 375g were generated by inserting blocks 1+2 and block 1 respectively, using PCR reaction with appropriate primers (Table S1), and construct 375a as template. Constructs 375b and 375f were generated by digesting construct 375a with BglII and HindIII. Construct 375c was generated by digesting plasmid 375a with StuI and HindIII. Construct 375e was generated by digesting plasmid 375a with StuI and BglII. For specific mutagenesis of the promoter, the insert from construct 375b was sub-cloned to pBS (Stratagene) creating plasmid pBS-375b. Plasmids bearing specific mutations were generated from construct pBS-375b using Quick™Change Site-Directed mutagenesis approach (Stratagene). All mutated inserts were fused to the firefly luciferase reporter gene in vector pGL3 using the unique sites NheI and XhoI.

### Cell culture

The following established cell lines were used in this study: HIT M2.2.2 (Hamster beta cells) [22], [23], βTC1 [24], and CHO (Chinese hamster ovary cells). Cells were grown in Dulbecco's modified Eagles medium (DMEM) supplemented with 10% fetal calf serum (FCS), penicillin (200 I.U./ml) and streptomycin (100 µg/ml).

### Transient transfections

Transfection experiments with HIT and CHO cells were carried out using the calcium phosphate co-precipitation technique [25]. Transfections were performed in either 10 cm or 6 well tissue culture plates (Falcon). The 10 cm plates contained 2×10^6^ cells (HIT cells) or 7×10^5^ cells (CHO) and the 6 well plates contained 4×10^5^ cells (HIT) or 2×10^5^ cells (CHO). When transfections were performed in 10 cm plates, the DNA mixture consisted of 2 µg reporter construct and 250 ng internal control plasmid. The total amount of DNA was equalized to 10 µg by adding pUC18. When transfections were performed in 6 well plates, the DNA mixture consisted of 0.5 µg reporter construct and 62.5 ng internal control plasmid. When expression plasmids were used, 1 µg of each was added. The total amount of DNA per transfection was equalized to 3 µg by adding pUC18. The precipitates were left on the cells for 4–7 h, and cells were then exposed to 20% glycerol (HIT) or 10% glycerol (CHO) in DMEM for 2 min. The glycerol was then diluted with PBS (Ca^2+^/Mg^2+^ free), removed, and fresh medium was added. Cells were harvested 48 h after transfection, and extracts were subjected to assays to determine the activity of reporter enzymes. Under these conditions, efficiency of transfection was typically 20–40% as determined by use of GFP reporter plasmids.

### Luciferase assays

Firefly luciferase and renilla luciferase assays were carried out as follows: whole cell extracts containing 5–50 µg (1–5 µl) of protein were added to 100 µl of either firefly luciferase assay buffer (20 mM Tricine, 0.1 mM EDTA, 1.07 mM (MgCO_3_)_4_Mg(OH)_2_*5H_2_O, 2.67 mM MgSO_4_, 3.3 mM DTT, 270 mM Coenzyme A, 470 mM Luciferin (Promega #E1602) and 530 mM ATP, pH 7.8.) or renilla luciferase assay buffer (0.1 M K_2_HPO_4_ and 0.1 M KH_2_PO_4_, pH 7.4, and 0.5 mM Coelenterazine (Calbiochem)). The samples were placed in a luminometer (LUMAC Biocounter M2500 or Modulus microplate, Turner Biosystems) and light output was determined over a 10 second interval. Firefly luciferase activity was normalized according to the activity of renilla luciferase.

### Transgenic mice containing a miR-375-EGFP construct

A plasmid (miR-375-EGFP) containing the mouse miR-375 gene upstream region (768 bp) was generated by replacement of the firefly luciferase open reading frame in pGL3-375a with the EGFP open reading frame (ORF) from the plasmid pEGFP-N1 (using XbaI and BglII sites). A DNA fragment (MluI-SalI) containing the miR-375 promoter and the EGFP ORF (devoid of vector sequences) was purified and microinjected to fertilized mouse oocytes. Microinjection was performed in the Weizmann Institute veterinary facility following standard protocols. Microinjected mouse embryos were transferred into the oviduct of foster females. Genotyping of tail tips was performed using the EGFP primers; top 5′ -AAGTTCATCTGCACCACCG- 3′ and bottom 5′- TCCTTGAAGAAGATGGTGCG -3′. This procedure yielded 4 independent transgenic mice expressing GFP.

### Immunofluorescence analysis of pancreas from miR-375-EGFP transgenic mice

Transgenic miR-375-EGFP mice were dissected and examined under an Olympus binocular microscope (SZX12) for GFP detection. The pancreas was then removed, washed in PBS and fixed in 4% PFA for 4 h at 4°C. Tissue was then incubated over-night in 70% ethanol and embedded in paraffin. Slides were de-paraffinized and subjected to antigen retrieval (10 mM sodium citrate, 0.5 mM citric acid, pH 6.0). Endogenous peroxidase activity was inhibited by incubation with hydrogen peroxide (3% H_2_O_2_ in 20% methanol). Slides were washed again with PBS and PBST (0.2% Triton X-100), blocked with CASblock (Zymmed laboratories), and then incubated overnight at 4°C with the following primary antibodies diluted in CASblock: rabbit anti-GFP 1∶100 (Molecular Probes, A6455), guinea pig anti-insulin 1∶200 (DAKO, A0564), mouse anti-somatostatin 1∶200 (Beta Cell Biology Consortium) and mouse anti-glucagon 1∶300 (Beta Cell Biology Consortium). Slides were then washed and secondary antibodies diluted in CASblock were applied for 1 h at room-temperature (Cy5 anti-rabbit, Cy3 anti-mouse and Cy2 anti-guinea pig). Slides were then washed again and stained with DAPI for nuclear staining. Finally, slides were mounted with aqueous mounting medium (IMMCO). For immunohistochemistry, slides were incubated with DAB substrate kit for peroxidase (Zymmed laboratories). Selective expression of GFP in pancreatic tissue was observed in all 4 transgenic mice examined. Quantitative co-expression data was obtained from a representative animal. Expression of GFP in insulin-positive cells was evaluated in 12 different fields ( 165 cells); expression of GFP in glucagon-positive cells was evaluated in 8 different fields (165 cells); expression of GFP in somatostatin-positive cells was evaluated in 7 different fields (90 cells).

### 5′ Rapid amplification of cDNA ends (5′-RACE)

First strand cDNA was synthesized from 5 µg of DNase-treated RNA prepared from HIT cells transfected with pGL3-375a construct, using reverse transcriptase (Affinity Script; Stratagene) according to the manufacturer's instructions. The primer used (bot. 375 NcoI, Table S1) was complementary to a sequence in the middle of block 4, located upstream to the firefly luciferase gene. The cDNA was purified using an PCR purification kit (RBC), and eluted in 40 µl DDW. A poly-G tail was added to the cDNA 3′ end using terminal deoxynucleotidyl transferase (TDT) (Promega) by incubating the purified cDNA with 1× TDT buffer (Promega), 0.83 mM dGTP and 20 U of enzyme at 37°C for 1 h. The reaction was stopped by heating to 65°C for 15 min. Following a second purification, cDNA was eluted in 50 µl of DDW. PCR was performed using the Expand high fidelity PCR system (Roche), with 5 µl of cDNA and 30 pmol of a reverse primer located nested to the primer used for reverse transcription, and a forward primer: GAATTC(C)_24_. PCR conditions were: 94°C 2 min; 30 cycles (of 94°C 30 sec, 60°C 30 sec, 72°C 3 min); followed by an additional incubation at 72°C for 5 min. The primer used for the PCR reaction was a bottom primer complementary to block 3 (Bot. block 3 BglII, Table S1). An aliquot of the PCR product was resolved on a 1% agarose gel. The resulting band was excised from the gel, purified, sub-cloned into pGEMT-Easy vector (Promega) and then sequenced. The transcription start site was identified as the sequence immediately adjacent to the poly C sequence.

### Statistical analysis

Analysis was performed using two way ANOVA. After establishing overall significance of the F-test, pairs of means were compared by the Tukey test (p = 0.05). InStat software (version 2.01) was used to evaluate standard error of the mean (SEM).

## Results

### Identification of conserved regions upstream of miR-375

**Figure 1:**
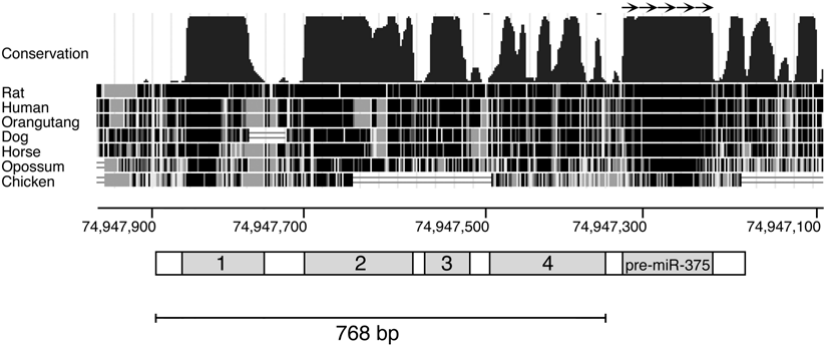
miR-375 gene regulatory region. Genomic locus of the mouse miR-375 gene and upstream conserved regions from UCSC browser (upper figure) with a graphic representation of the conserved regions upstream of the pre-miR-375 sequence (lower figure). The location of conserved blocks 1–4, and pre-miR-375 is indicated.

Important control elements can often be discovered by a careful inspection of the genomic sequence conservation among different species [26]. Therefore, we compared the region upstream of the pre-miR-375 sequence (miRBase accession # MI0000792) among several vertebrate orthologs. We identified four highly conserved sequences, which we named blocks 1–4 (Fig. 1). Block 1 is 156 bp long, is 59% identical between mouse and human, and is located 585 bp upstream from the pre-miR. Block 2 is 190 bp long, and is 66% identical between mouse and human. Block 3 is 71 bp long, and is 75% identical between mouse and human, and block 4 is 149 bp long, with 75% identity between mouse and human.

### Function of pri-miR-375 promoter in vivo

**Figure 2:**
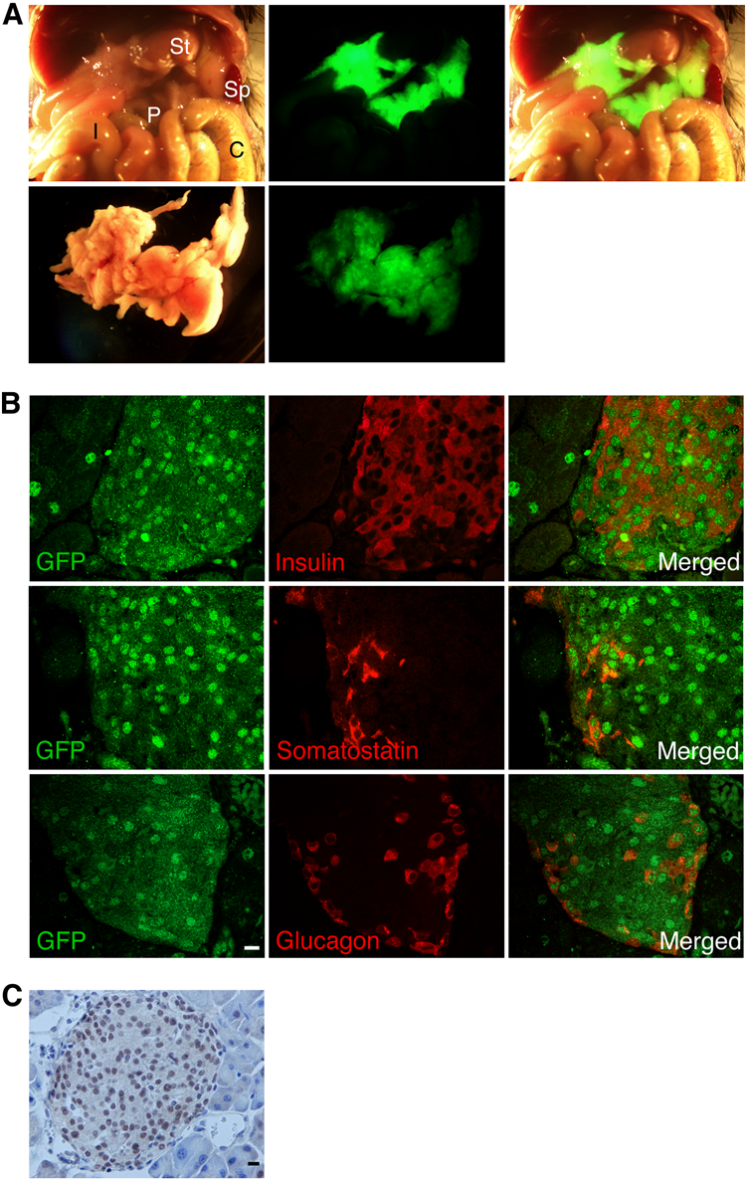
Expression of GFP in pancreas and islets of miR-375-EGFP transgenic mice. A. Abdominal organs of a 6 week old transgenic mouse (bright field - upper left, GFP - upper middle, merged -upper right. I, intestine. C, colon. P, pancreas. Sp, spleen. St, stomach). The pancreas was dissected and photographed (bright field - lower left, GFP – lower right). B. Immunofluorescence analysis of pancreas sections showing GFP (green), and insulin, somatostatin or glucagon (red). C. Immunohistochemical analysis using anti-GFP antibodies, counter-stained with hematoxylin. Scale bar indicated in B and C represents 10 µm.

In order to test the ability of these sequences to control gene expression *in vivo*, we fused a 768 bp DNA fragment containing the four conserved blocks (Fig. 1) to the GFP gene. The construct was microinjected into mouse oocytes and expression of GFP was monitored in developing embryos and adult transgenic mice. Strong GFP expression was seen in the pancreas of adult mice (Fig. 2A) and immunofluorescence microscopy revealed GFP expression within the islets (Fig. 2B). GFP was expressed in beta cells (97% of beta cells co-express GFP), in alpha cells (89% co-expression) and in delta cells (91% co-expression) (Fig. 2B). Immunohistochemical analysis confirmed that GFP is expressed at high levels in pancreatic islet cells, and at much lower levels in pancreatic exocrine cells (Fig. 2C). A small number of GFP positive cells were found scattered fairly evenly throughout the exocrine pancreas (Fig. 2B,C). We examined the expression of GFP in 4 mice, each derived from an independent microinjected oocyte. The selectivity of expression for pancreatic tissue was observed in all 4 mice (data not shown). Since this pattern of expression in different animals presumably results from independent insertions into random genomic loci, we conclude that the region upstream of miR-375 contains the pri-miR-375 gene promoter, and is capable of directing GFP expression selectively to pancreatic islets in vivo.

### Characterization of the pri-miR-375 transcription start site

**Figure 3:**
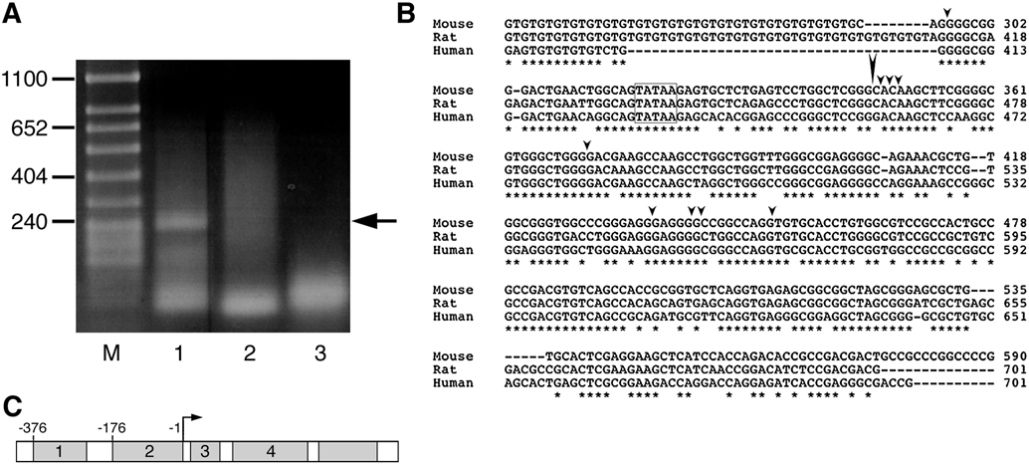
Mapping of transcription start site of miR-375 gene by 5′-RACE. A. DNase-treated total RNA from HIT cells transfected with the pGL3-375a construct was reverse transcribed, using a primer complementary to the luciferase sequence. The cDNA was poly-(dG) tailed and amplified by PCR using a poly-C primer and a nested primer complementary to block 4 (lane 1). Lane 2, no RT control; lane 3, no template control. Marker sizes are indicated. The location of the band in lane 1 is indicated by an arrow. B. Alignment of miR-375 upstream sequences (from −96 to +244, relative to transcription start site) of mouse, rat and human. The primer used for PCR amplification is located at the middle of block 4. The TATA sequence is marked by a box. The large arrow-head indicates the major transcription start site. Small arrow-heads indicate the start site of shorter species detected by 5′-RACE. C. Representation of the pGL3-375a construct. Conserved regions 1–4 are indicated. The luciferase gene is indicated as an unmarked open box. The arrow indicates the transcription start site revealed by 5′-RACE analysis. Numbers above the bar indicate location relative to the transcription start site.

In order to confirm that the 768 bp upstream of miR-375 contains a functional promoter, we wished to identify the transcription start site of the endogenous pri-miR-375 transcript. Using the 5′-RACE procedure with mRNA derived from the beta cell line βTC1 [24], we were unable to identify a discrete band corresponding to the start site of the endogenous pri-miR-375, presumably because of rapid processing of the precursor molecule [27]. As an alternative approach, we performed 5′-RACE using RNA derived from the beta cell line βTC1 transfected with a plasmid containing the 768 bp upstream region linked to the luciferase reporter gene. For the PCR reaction, we used a primer corresponding to a sequence located in the middle of block 4. A discrete band was obtained from this reaction (Fig. 3A, lane 1), whereas this band was not seen in control reactions (Fig. 3A, lanes 2–3). The resulting band was analyzed by DNA sequencing. The sequence indicated a transcription start site 259 bases upstream of the pre-miR-375 start (marked with large arrowhead in Fig. 3B, and an arrow in Fig. 3C). Strikingly, this putative start site is 24 bases downstream of an evolutionarily conserved TATA box located at the 3′ end of conserved block 2. Additional 5′-RACE experiments performed using primers distributed in the miR-375 regulatory region identified the same start site (not shown). Although in some sporadic cases, additional bands were obtained (marked with small arrowheads in Fig. 3B), sequence analysis showed that they were either non-relevant (sequences unrelated to miR-375) or shorter species, most likely resulting from premature polymerase termination caused by high GC content, or stable secondary structure of the pri-miR-375. These data suggest that transcription of the pri-miR-375 indeed initiates 24 bases downstream of the conserved TATA box, and that the conserved blocks 1 and 2 contain the promoter of the miR-375 gene.

### Characterization of the pri-miR-375 promoter

**Figure 4:**
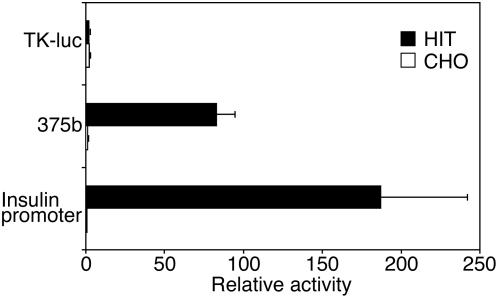
Activity of the miR-375 promoter in cultured cells. The putative promoter region of the miR-375 gene (construct 375b) was ligated upstream to the firefly luciferase reporter gene in the promoter-less pGL3-basic vector. As a positive control, the insulin promoter was fused upstream to the firefly luciferase reporter gene. The herpes simplex virus thymidine kinase (TK) promoter fused upstream to the firefly luciferase reporter gene served as non cell-specific control promoter. Promoter activity of each construct was determined following transfection to the beta cell line HIT or the non-beta cell line CHO. Values were normalized for transfection efficiency according to the activity of a co-transfected renilla luciferase plasmid and expressed relative to the activity of pGL3-basic vector. Each data point represents the mean±SEM of at least three independent transfection experiments.

To functionally characterize the miR-375 promoter, the fragment consisting of blocks 1 and 2 was fused to a firefly luciferase reporter gene and transfected into pancreatic beta and non-beta cell lines (Fig. 4, fragment 375b). For comparison, we used the promoter of the herpes simplex virus thymidine kinase (TK) gene, which shows low constitutive activity in many cells types [23]. The fragment 375b directed 32-fold higher luciferase gene expression than the TK promoter in beta (HIT) cells (Fig. 4). On the other hand, activity of the fragment was weaker than the TK promoter in CHO cells. Preferential activity of the pri-miR-375 promoter was also seen on comparison with an additional non-beta cell line NIH-3T3 (data not shown). Thus the fragment indeed possesses promoter activity that is manifested preferentially in beta cells. Indeed, the promoter activity of construct 375b was remarkably high, since it showed approximately 45% of the activity of the insulin promoter (Fig. 4), which is considered to be a very strong beta cell promoter [15]. Consistent with the activity observed in mouse pancreatic alpha cells (Fig 2B), construct 375b also showed high activity (approximately 20-fold higher than the TK promoter) in the alpha cell line αTC1 (data not shown).

**Figure 5:**
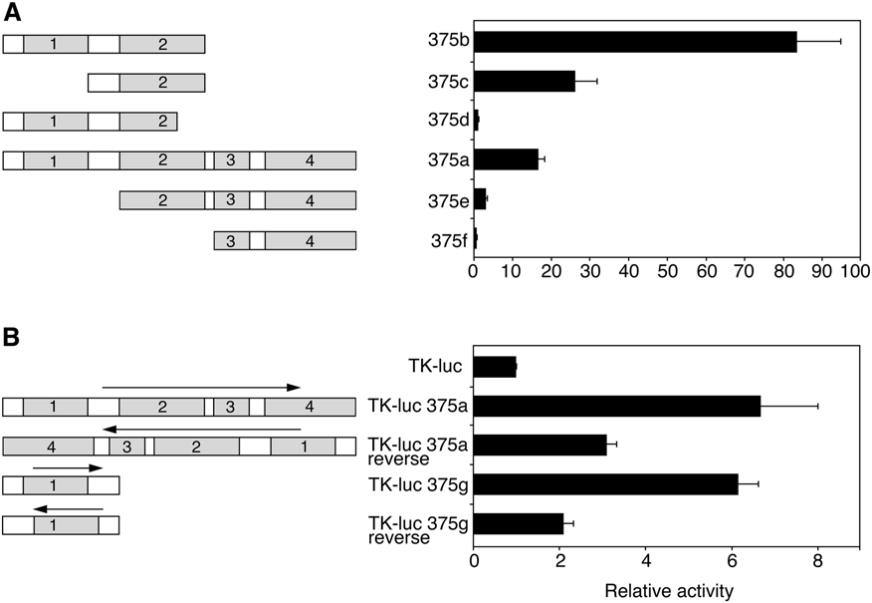
Mapping of transcriptional regulation regions by deletion analysis. A. Portions of the miR-375 upstream region were deleted according to the location of the conserved blocks (Fig. 1) and ability to drive expression of firefly luciferase reporter gene was determined in the context of the promoter-less pGL3-basic vector. Values shown are mean±SEM of at least 3 independent transfection experiments. B. Construct 375a or 375g were tested for enhancer activity. The different regions were ligated (in both orientations) upstream to the TK promoter driving expression of the firefly luciferase reporter gene. Normalised luciferase activity was expressed relative to the activity of pGL3-basic vector (A) or TK-LUC (B). Values shown are mean±SEM of at least 3 independent transfection experiments.

To determine the contribution of the conserved sequence blocks to the activity of the 375b fragment, deletion analysis was performed (Fig. 5A). Removal of block 1 (construct 375c) led to 3-fold reduction in activity, while beta cell specificity was maintained in this construct (data not shown). Deletion of the ∼100 bp at the 3′ end of block 2, containing the conserved TATA box, led to a dramatic (75-fold) reduction in activity (construct 375d). Unexpectedly, the presence of conserved blocks 3 and 4 in the context of blocks 1+2 or block 2, reduced activity substantially, suggesting that blocks 3 and 4 may contain a negatively-acting element. Taken together, these data show that blocks 1 and 2 contain the promoter of the pri-miR-375 gene, and that the TATA box area is critical for promoter activity.

Since mammalian transcriptional control regions often contain transcriptional enhancers, we tested the ability of the upstream miR-375 sequences to activate transcription from the heterologous promoter TK. Indeed fragment 375a (corresponding to blocks 1–4) was able to activate the TK promoter 6-fold in HIT cells (Fig. 5B) but showed essentially no effect on the TK promoter in non-beta cells (CHO) (data not shown). Substantial activation was still maintained upon inversion of the fragment relative to the promoter (Fig. 5B). Similar results were obtained with block 1 (Fig. 5B). We conclude that sequences in the conserved region within block 1 possess features characteristic of a transcriptional enhancer.

### Mutational analysis of block 1 and block 2

**Figure 6:**
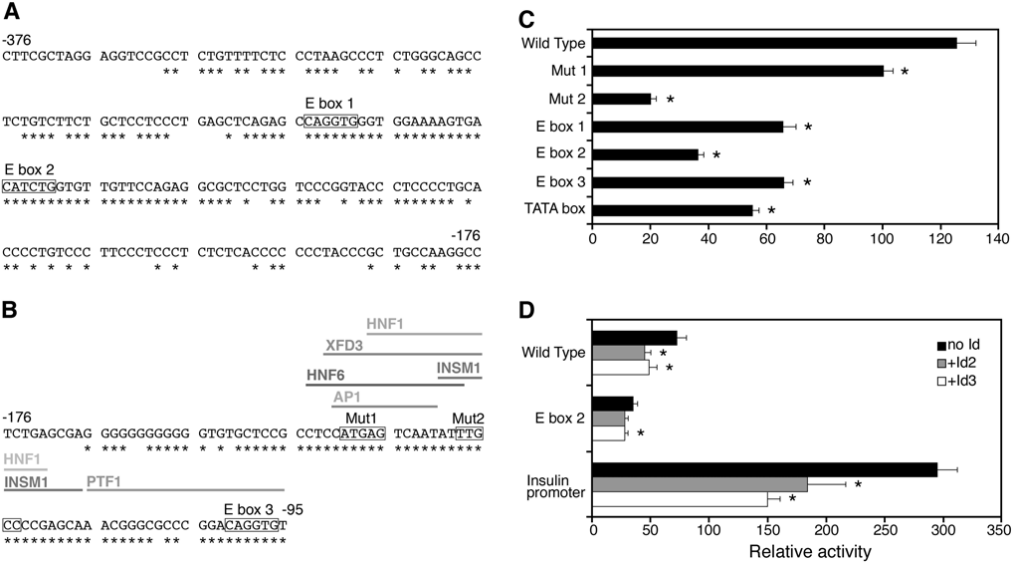
Mutagenesis of putative transcription factor-binding sites. A and B. Nucleotide sequence of conserved regions of mouse miR-375 promoter. Stars indicate nucleotides conserved among mouse, rat, and human miR-375 genes. Location relative to the transcription start site is marked by numbers at the beginning and end of the sequence. A. Alignment of Block 1. E boxes are indicated. B. Alignment of block 2. Sites of binding sites for putative transcription factors, E boxes, and mutations are marked on sequence. C. Specific sites were mutated and reporter plasmids containing the mutations were tested for luciferase activity as before. Values shown are mean±SEM of at least 4 independent transfection experiments. *, p<0.05, as compared with wild-type. D. The indicated constructs were transfected to HIT cells in the presence or absence of Id2 or Id3 expression vectors. Normalised luciferase activity was expressed relative to the activity of pGL3-basic vector. Values shown are mean±SEM of at least 4 independent transfection experiments. *, p<0.05, as compared with transfections performed in the absence of Id expression vector.

It has previously been shown that E-boxes (consensus sequence CAxxTG) play an important role in directing selective expression of beta cell specific promoters [20]. Indeed three conserved E-box elements are present in the promoter sequence (Fig. 6A,B). We mutated each of these E-boxes and measured luciferase activity following transfection to HIT cells. Mutation of E-boxes 1, 2 and 3 led to reductions of 47%, 71% and 47% of activity, respectively, as compared to the wild type construct (Fig. 6C). Likewise, mutation of the TATA box caused a 56% reduction in activity (Fig. 6C). Bioinformatics analysis using the MatInspector program (Genomatix) revealed potential binding sites for several beta-cell transcription factors: HNF1, HNF6, AP1, INSM1, XFD3 and PTF1, within the most conserved region of block 2 (Fig. 6B). Mutation 1 (perturbing the consensus binding site for AP1, XFD3, and HNF6) led to a small loss of activity (reduction of 20%), whereas mutation 2 (perturbing the consensus binding site for INSM1, and HNF1) led to a much larger loss in activity (reduction of 84%). This experiment therefore identifies multiple cis-elements required for full activity of the miR-375 promoter.

To further examine the idea that the conserved E-boxes of the miR-375 promoter act as binding sites for transcription factors of the bHLH family, we tested the effects of the dominant negative HLH proteins Id2 and Id3 [28]. As a control, we first verified that both Id2 and Id3 expression vectors were able to inhibit insulin promoter activity, showing that the constructs were functional under the experimental conditions (Fig. 6D). Both Id2 and Id3 significantly repressed the activity of the miR-375 promoter (38% and 33% respectively, Fig 6D) as compared to transfections performed in the absence of Id-encoding plasmids. Consistent with this, the effect of the Id proteins on the promoter fragment mutated in one of the E-boxes (E-box 2), was less pronounced. These results suggest that the E-boxes within the promoter are functioning at least in part by interaction with transcription factors of the bHLH family.

## Discussion

miRNAs are emerging as critical regulators of a broad range of developmental processes [1]. In order to understand how miRNAs function, major efforts have focused on the identification of *bona fide* target genes. For example, miRNAs have recently been shown to regulate expression of the pancreatic developmental factors Ngn3 and Foxa2 [29]–[31]. On the other hand, the appreciation that many miRNAs show highly selective patterns of expression underscores the importance of elucidating the mechanisms that regulate miRNA biogenesis, which is currently poorly understood. For protein coding genes, the central role of transcription initiation in regulating expression levels is well established. However, in recent years, it has become clear that a wide variety of post-transcriptional mechanisms can also significantly affect expression. The aim of this study was to determine whether transcriptional control plays a significant role in directing cell-specific expression of the pri-miR-375 gene which is expressed selectively in pancreatic islets. Using functional assays, we have been able to show that an evolutionarily conserved region upstream of the gene can confer selective expression of a reporter gene, both in transgenic mice and in transfected tissue culture cells. Deletion analysis identified a number of conserved cis-elements required for optimal activity, including a TATA sequence. Consistent with this, we have identified the major transcription start site 24 bp downstream of the TATA sequence. These data represent the first characterization of a pancreas-specific miRNA gene promoter, and demonstrate that cell-specific expression is regulated at least in part, at the level of transcription. This does not exclude possible involvement of post-transcriptional control mechanisms, and indeed a recent study raises the possibility that expression of miR-375 in the developing endocrine pancreas may be controlled in part by selective processing [32].

Our study provides some indications of transcription factors that may be involved in regulating the activity of the pri-miR-375 promoter. One region within block 2 that was particularly sensitive to mutation contains consensus binding sequences for the factors HNF1 and INSM1, which have been previously implicated in development and function of pancreatic islets [33]. Our mutational analysis also showed that E boxes are required for full transcriptional activity. This raises the possibility that the miR-375 gene may be regulated by bHLH transcription factors such as Ngn3 and NeuroD1, which are known to play a central role in pancreas endocrine development and in mature beta cell function respectively [33]. Indeed, recent chromatin immunoprecipitation experiments have shown that NeuroD1 interacts with conserved sequences both upstream and downstream of the miR-375 gene [34]. In the same study, the key pancreatic transcription factor Pdx-1 was also shown to interact with the upstream region, raising the possibility that these factors cooperate in activating transcription, as has been found with other beta cell-specific promoters e.g. the insulin gene promoter. Interestingly, this region contains no Pdx-1 consensus target sites, suggesting that the action of Pdx-1 may be indirect. Taken together, these results suggest that selective expression of miR-375 is controlled by a number of transcription factors that participate in the transcriptional cascade that shapes pancreatic development, and is therefore consistent with the possibility that miR-375 itself is a component of this cascade

Thus far, relatively few potential targets of miR-375 have been experimentally validated [11]. Recently, PDK1, a mediator of the PI3K/PKB signaling cascade, was identified as a potential target of miR-375; in the same study, glucose was shown to inhibit production of miR-375 [35]. Since activation of the PI3K cascade can lead to increased beta cell proliferation, these finding may represent a mechanistic link between glucose and beta cell proliferation. This may be of considerable physiological importance, since glucose has been proposed as a potential mediator of beta cell hyperplasia in insulin resistant states such as obesity and pregnancy [36]. The possibility that glucose modulates miR-375 expression through regulation of promoter activity needs to be further explored.

The large number of miRNA genes combined with the potential of each miRNA to regulate multiple target mRNAs, implies a regulatory network of great complexity. This is consistent with the pleiotropic effects observed in numerous studies, such as those involving global inhibition of miRNA production by ablation of the Dicer gene [14], [37]. On the other hand, experiments involving loss of function of miRNAs often show surprisingly mild phenotypes [38], [39]. To resolve this apparent paradox, it has been proposed that miRNAs may act to confer robustness on genetic programs [40]. Thus, miRNAs may repress “leaky” expression of genes that are not required in a particular biological setting [41]. Alternatively, miRNAs may be required to buffer stochastic variations in expression of genes An important feature of these models is the hypothesis that key transcription factors may regulate target genes both directly and indirectly, through modulation of expression of miRNAs [42].

It is becoming apparent that miRNAs are integral components of transcriptional regulatory networks underlying the development and maintenance of differentiated cell types. The identification of miR-375 as a likely target for key pancreatic transcription factors further strengthens the emerging notion that miRNAs are involved in regulatory networks controlling pancreatic development. More detailed analyses of the transcriptional control mechanisms controlling miR-375 and other selectively expressed miRNA genes will help to shed light on these networks, and permit a more detailed understanding of many aspects of cell function in both physiological and pathological states.

## Supporting Information

Table S1 Supplementary data
(0.05 MB DOC)
