# Supplementary material for: The Promoter of the pri-miR-375 Gene Directs Expression Selectively to the Endocrine Pancreas
Source: PLoS One. 2009 Apr 3;4(4):e5033. doi: 10.1371/journal.pone.0005033 (PMC2660411; doi:10.1371/journal.pone.0005033)
Supplement: Table S1 — Supplementary data (0.05 MB DOC) [file pone.0005033.s001.doc]

**Table S1. Primers used**

| **Name and details** | **Seq. 5’-3’** |
| --- | --- |
| top 375 bgl2 | GAAGATCTTGAGGTACATCGCAGAGGCCAG |
| bot 375 nco1 | CATGCCATGGGGGCCGGAGCGGAAGACCC |
| bot block 1 BglII | GAAGATCTTCGCTCAGAGGCCTTGGCAGC |
| top block 2 BglII | GAAGATCTAGGCCTCTGAGCGAGGGG |
| bot block 2 BglII | GAAGATCTCACACACACCTGTCCGGG |
| top block 3 BglII | GAAGATCTTGCAGGGGCGGGGACTGA |
| bot block 3 BglII | GAAGATCTCTCGAGTGCACAGCGCTC |
| top block 4 BglII | GAAGATCTGAAGCTCATCCACCAGACAC |
| bot block 4 BglII | GAAGATCTGGGCCGGAGCGGAAGACC |
| bot1 pBS | GATGAACGAAATAGACAGATCG |
| Top2 pBS | CGATCTGTCTATTTCGTTCATC |
| Mutation 1 TOP | GGTGTGCTCCGCCTCCTCGAGTCAATA  TTTGCCCCG |
| Mutation 1 BOT | CGGGGCAAATATTGACTCGAGGAGGCG  GAGCACACC |
| Mutation 2 TOP | GCCTCCATGAGTCAATATGTGCACCGAG  CAAACGGGCGCCCG |
| Mutation 2 BOT | CGGGCGCCCGTTTGCTCGGTGCACATA  TTGACTCATGGAGGC |
| Mutation 3 TOP | CAATATTTGCCCCGAGCAAACGGGCGC  CCGGGACCCGTTGTGTG |
| Mutation 3 BOT | CACACAACGGGTCCCGGGCGCCCGTTT  GCTCGGGGCAAATATTG |
| Mutation 4 TOP | GCTCCTCCCTGAGCTCAGAGCACTAGT  GGTGGAAAAGTGACATCTGG |
| Mutation 4 BOT | CCAGATGTCACTTTTCCACCACTAGTG  CTCTGAGCTCAGGGAGGAGC |
| Mutation 5 TOP | GGTGGGTGGAAAAGTGACGCGTGTGTT  GTTCCAGAGGCGCTCC |
| Mutation 5 BOT | GGAGCGCCTCTGGAACAACACACGCGT  TCACTTTTCCACCCACC |
| Mutation TATA TOP | GGGGACTGAACTGGCAGGCGCCGAGTG  CTCTGAGTCCTGG |
| Mutation TATA BOT | CCAGGACTCAGAGCACTCGGCGCCTGC  CAGTTCAGTCCCC |
| Block3 top StuI | GAAGGCCTTGCAGGGGCGGGGACTGA |
| Block 4 bot StuI | GAAGGCCTGGGCCGGAGCGGAAGACC |
| Block 3 top TG7 BglII | GAAGATCTTGTGTGTGTGTGTGCAGGG |
| Block3 top HindIII | CCCAAGCTTTGCAGGGGCGGGGACTGA |
| Block4 BOT HindIII | CCCAAGCTTGGGCCGGAGCGGAAGACC |
